# Directional Confusions Reveal Divergent Inductive Biases Through Rate-Distortion Geometry in Human and Machine Vision

**Published:** 2026-05-14

**Authors:** Leyla R. Caglar, Pedro A.M. Mediano, Baihan Lin

**Affiliations:** Windreich Department of AI and Human Health, Icahn School of Medicine, at Mount Sinai, New York, NY, USA; Department of Computing, Imperial College London, London, UK; Windreich Department of AI, and Human Health, Department of Psychiatry, Icahn School of Medicine, at Mount Sinai, New York, NY, USA

## Abstract

To humans, a robin seems more like a bird than a bird seems like a robin, but does this asymmetry also hold for machine vision? Humans and modern vision models can match each other in accuracy while making systematically different kinds of errors, differing not in how often they fail, but in who gets mistaken for whom. We show these directional confusions reveal distinct inductive biases invisible to accuracy alone. Using matched human and deep neural network responses on a natural-image categorization task under 12 perturbation types, we quantify asymmetry in confusion matrices and link its organization to the geometry of the information–error trade-off — how efficiently, and how gracefully, a system generalizes under distortion. We find that humans exhibit broad but weak asymmetries across many class pairs, whereas deep vision models show sparser, stronger directional collapses into a few dominant categories. Robustness training reduces overall asymmetry magnitude but fails to recover this human-like distributed structure. Generative simulations further show that these two asymmetry organizations shift the trade-off geometry in opposite directions even at matched accuracy, explaining why the same scalar asymmetry score can reflect fundamentally different generalization strategies. Together, these results establish directional confusion structure as a sensitive, interpretable signature of inductive bias that accuracy-based evaluation cannot recover.

## 1 Introduction

People judge a robin to be more similar to a bird than a bird is to a robin. An ellipse seems more like a circle than a circle seems like an ellipse [Tversky and Gati, 1982]. These directional asymmetries are not noise, they reflect the graded structure of cognitive representations — prototype effects [Rosch, 1975], feature salience [Tversky, 1977], category typicality [Rosch and Mervis, 1975] — and they violate the assumptions of symmetric similarity spaces [Shepard, 1964, 1987]. Directional confusions in categorization tasks [Nosofsky, 1986, 1991, Getty et al., 1979, Kahana and Sekuler, 2002] can therefore serve as diagnostic signatures of representational bias and efficiency [Sims, 2018, Jakob and Gershman, 2023].

Do artificial vision systems exhibit the same asymmetric structure or a fundamentally different one? Humans and modern ANNs increasingly reach similar categorical accuracy, but most evaluation pipelines treat confusion as unstructured, asking how often a system errs rather than who gets mistaken for whom and in which direction [Gupta et al., 2021, Attarian et al., 2020]. Yet directional structure is precisely where inductive biases leave their fingerprint, reflecting the priors a system implicitly imposes when mapping ambiguous or degraded inputs to categories.

However, evaluations of ANNs under distribution shift typically abstract away error structure. Metrics such as accuracy or top-*k* error implicitly treat confusion patterns as symmetric or irrelevant [Geirhos et al., 2020, Recht et al., 2019], even though deep vision models can produce highly structured one-way failures, such as collapsing diverse subcategories into a dominant prototype (e.g., many dog breeds into “Labrador”) without the reverse confusion occurring [Shankar et al., 2020, Miller et al., 2021, D’Amato et al., 2025]. Enhanced under adversarial noise or texture-based corruptions, models may confuse texture-diagnostic categories (e.g., “zebra” with “barcode”) in one direction only [Geirhos et al., 2018a, Ilyas et al., 2019]. Large-scale evaluations show that this gap between human and machine error consistency persists even in state-of-the-art models [Geirhos et al., 2021], and simplicity bias — the tendency of ANNs to rely on the simplest predictive features available — provides a mechanistic account of why such structured failures arise [Shah et al., 2020]. The most direct precedent, Liu et al. [2025], compares human and machine confusion matrices at the level of aggregate error distributions, but does not characterize the directional organization of asymmetries or link error structure to generalization properties.

This raises a targeted question: when vision systems are matched for task, perturbation, and accuracy level, do humans and ANNs exhibit systematically different directional confusion structure, and does that structure predict generalization geometry independently of accuracy? Cognitive accounts explain human asymmetries via graded similarity and attentional biases [Tversky, 1977, Rosch, 1975], predicting broad but weak directional tendencies spread across many class pairs. By contrast, prototype collapse and shortcut-driven feature reliance in ANNs [Shankar et al., 2020, Geirhos et al., 2018b, Shah et al., 2020] predict sparser but stronger sink-like failures concentrated on a few classes — representations collapsed onto a small set of dominant decision boundaries rather than the graded similarity gradients that characterize human-like representations [Sorscher et al., 2022, Wei and Woodford, 2025]. These two organizations reflect fundamentally different priors. Distributed asymmetries indicate sensitivity to graded similarity across many feature dimensions, while sink-like collapses indicate rigid reliance on a small set of dominant decision boundaries. Critically, two systems can show similar accuracy (or even similar aggregate asymmetry) while failing for qualitatively different reasons, with different implications for downstream reliability. We therefore not only compare asymmetry structure across systems, but explicitly test whether the organization of directional errors carries information about generalization that is independent of accuracy.

To formalize this, we adopt a rate–distortion (RD) framework that treats each system as a noisy communication channel and summarizes its generalization behavior as a geometric signature of the information–error trade-off — how efficiently, and how gracefully, a system compresses stimulus–response information under distortion (see Section 2). Within this formalism, the *organization* of asymmetry, not just its magnitude, predicts where a system sits on the RD frontier, linking the structure of directional errors directly to generalization geometry. We ask whether humans and ANNs dissociate in asymmetry organization, how that structure relates to generalization geometry independently of accuracy, and whether robustness training closes the gap. We find that:

- Humans distribute confusion broadly and weakly across many class pairs; ANNs concentrate failures into a few strong, sink-like collapses — a dissociation invisible to accuracy.
- This organizational difference predicts generalization geometry independently of accuracy: the same performance can mask fundamentally different failure regimes.
- Generative simulations confirm the two organizations produce opposite effects on generalization geometry even at matched accuracy, explaining why scalar asymmetry metrics are insufficient.
- Robustness training reduces overall asymmetry but leaves the organizational dissociation intact.

## 2 Rate-Distortion Framework

**Figure 1:**
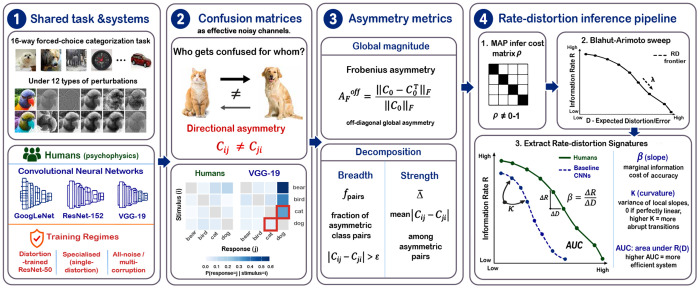
Pipeline overview. The full analysis pipeline, from (1) matched human and ANN stimulus–response behavior under perturbation, (2) confusion-matrix construction, illustrating contrast between human broad-weak errors and ANN sink-like collapses (noise perturbation condition (σ = 0.10)), to (3) schematic asymmetry decomposition into breadth and strength, and (4) schematic RD frontier inference yielding three geometric signatures (*β*, *κ*, AUC).

To formalize how directional error structure relates to generalization, we adopt a rate–distortion (RD) framework grounded in information theory and efficient coding [Shannon, 1948, Sims, 2018]. We treat each system - human or machine - as an effective noisy communication channel whose behavior is summarized by its stimulus–response confusion matrix. The central object is the information–error trade-off: how much mutual information between stimulus and response must be preserved to achieve a given level of categorical distortion. Tracing this trade-off yields a rate–distortion (RD) frontier [Shannon, 1948], and the shape of that frontier characterizes how efficiently and how gracefully a system generalizes under compression [Sims, 2018] (see Fig. 1 for an illustrated inference pipeline).

Concretely, we infer a latent distortion matrix $\rho \in R_{\geq 0}^{K\times K}$ from each observed confusion matrix via maximum-a-posteriori (MAP) estimation, using Blahut–Arimoto (BA) iterations [Blahut, 1972, Arimoto, 1972] to trace the optimal channel at each compression level. Sweeping an inverse-temperature parameter λ over the resulting frontier yields three compact geometric signatures:

- **Slope** (*β*): the median finite-difference derivative Δ*R*/Δ*D* along the frontier, capturing the marginal information cost of reducing expected error.
- **Curvature** (*κ*): the variance of local slopes, capturing how nonuniform the trade-off is across operating points — higher *κ* indicates more abrupt, threshold-like transitions.
- **Efficiency** (AUC): the trapezoidal area under *R(D)* across the swept λ range, summarizing overall frontier geometry rather than performance at any single compression level.

A key property of this framework is that it accommodates asymmetric confusion matrices directly, without forcing behavioral data into a symmetric similarity space. This makes it a natural formalism for the present analysis. The RD frontier is shaped by the full structure of the distortion matrix — including its asymmetric component — so systems with different asymmetry organizations are expected to trace qualitatively different frontiers, even at matched accuracy levels. The organization of directional errors, not just their magnitude, is therefore a first-class input to generalization geometry (Fig. 1, Boxes 2-4).

## 3 Methods and Modeling Framework

### 3.1 Datasets, Perturbations, and Evaluated Systems

We analyze matched human and ANN model behavior on controlled perturbations of natural images in a *K* = 16 ImageNet-derived categorization setting. The primary stimulus benchmark is the Generalization repository (GEN; [Geirhos et al., 2018b]), which includes twelve perturbation families (e.g., colour/grayscale, contrast, filtering, phase noise, power equalisation, rotation, Eidolon variants, and uniform noise), each parameterized by distortion strength to produce systematic out-of-distribution (OOD) conditions. The data includes ~83k human psychophysics trials, as well as three baseline pretrained convolutional neural networks (CNNs): GoogLeNet Szegedy et al. [2015], ResNet-152 He et al. [2016], and VGG-19 Simonyan and Zisserman [2014]. To study training-induced variation, we evaluate models with different robustness regimes. In particular, we include (a) *Distortion-trained* ResNet-50 models trained from scratch with distorted training distributions, (b) *Specialised* single-distortion models trained on one perturbation family and evaluated across all perturbations, and (c) *All-noise / multi-corruption* models trained on mixtures of noise-like corruptions and evaluated across individual perturbations (see the GEN repository [Geirhos et al., 2018b] for further details on benchmark stimuli, model training, and task evaluations.

### 3.2 Confusion Matrices as Effective Behavioral Channels

We treat each system’s confusion matrix as defining an effective noisy channel, then ask what latent distortion structure — and what information–error trade-off — is implied by its pattern of errors, including their directional asymmetries (Fig. 1, Box 2). For each system *s*, experiment *e*, and distortion level *d*, we summarize stimulus–response behavior with a *K* × *K* confusion matrix *N^(s,e,d)^*, where *N_ij_* counts responses of class *j* to stimuli of class *i*. Row-normalization yields an empirical conditional distribution $C_{ij}^{\left(s,e,d\right)}={Pr}_{s}(y=j|x=i;e,d)\approx N_{ij}^{\left(s,e,d\right)}/\sum_{j^{'}}N_{ij^{'}}^{\left(s,e,d\right)}$, where $C_{0}^{\left(s,e,d\right)}$ denotes $C^{\left(s,e,d\right)}$ with its diagonal entries set to zero (i.e., excluding correct responses). Each system is treated as a noisy communication channel from stimulus *x* to response *y*, and we infer a latent distortion matrix *ρ* ∈ $\rho \in R_{\geq 0}^{K\times K}$ using maximum-a-posteriori (MAP) estimation. The likelihood is evaluated via the BA-optimal channel [Blahut, 1972, Arimoto, 1972] under *ρ* (with scale absorbed into *ρ*), following the RD fitting procedure of Sims [2018], as described in Section 2, and adapted here to analyze directional asymmetry.

To trace the rate–distortion (RD) frontier, we scale *ρ* by an inverse-temperature parameter λ > 0 and compute the corresponding optimal channel. Specifically, we solve for the RD-optimal channel $q_{\lambda }(y|x)$ via Blahut–Arimoto fixed-point updates: $q_{\lambda }(y|x)\propto p(y)exp(-\lambda \rho (x,y))$, iterated jointly with $p(y)=\sum_{x}p(x)q_{\lambda }(y|x)$ until convergence.

We then trace the RD frontier over a log-spaced grid of λ values (e.g., λ ∈ [10^−1^, 10^3^]) and extract three compact RD signatures: slope *β* (median finite-difference Δ*R*/Δ*D* along the frontier; see Appendix A4.1 for boundary conditions), curvature *κ* (variance of local slopes), and efficiency AUC (trapezoidal area under *R(D)* over the swept λ range). We use AUC rather than point-wise distance to the RD curve because it integrates efficiency across all operating points, providing a summary of the full frontier geometry rather than performance at a single compression level.

### 3.3 Quantifying Asymmetry

We define directional asymmetry as deviation from matrix symmetry in the row-normalized confusion matrix C, with a threshold $\varepsilon =10^{-12}$ to suppress numerical noise (Fig. 1, Box 3). We use three complementary measures. **Breadth**: fpairs=npairs/K2, where $n_{\text{pairs}}=\sum_{i<j}I\left[\left|C_{ij}-C_{ji}\right|>\varepsilon \right]$, captures how many class pairs exhibit any directional asymmetry. **Strength**: $\overset{‾}{\Delta }=E_{i<j}\left[\left|C_{ij}-C_{ji}\right||\right.\left.\left|C_{ij}-C_{ji}\right|>\varepsilon \right]$ captures the mean magnitude of directional deviation among asymmetric pairs, where $E_{i<j}[\cdot ]$ denotes the sample mean over all ordered pairs i<j. **Global magnitude**: quantified by the normalized off-diagonal Frobenius norm $A_{F}^{\text{off}}(C)={\left\|C_{0}-C_{0}^{⊤}\right\|}_{F}/{\left\|C_{0}\right\|}_{F}$, which excludes correct responses from both numerator and denominator and is used as the asymmetry predictor in all analyses reported here. Implementation details and the choice of ε are in Appendix A1.1. All analyses use block-wise aggregates (experiment × condition × model instance) to avoid pseudo-replication. We compare groups using Wilcoxon rank-sum tests for robustness to non-normality and Welch’s t-tests for effect size estimation, with p-values corrected via Benjamini–Hochberg FDR (BH–FDR) and 95% CIs via the Welch–Satterthwaite approximation.

### 3.4 Linking Asymmetry to RD Geometry

We test whether directional confusability covaries with RD behavior (Fig. 1, **Box 4**). Because the RD frontier is shaped by the full structure of the distortion matrix — including its asymmetric component — systems with different asymmetry organizations are expected to trace qualitatively different frontiers, even at matched accuracy levels. To quantify this, our primary asymmetry measure is the *normalized off-diagonal Frobenius asymmetry* computed from the row-normalized confusion probabilities. For each block, let *C* denote the row-normalized confusion matrix and *C*_0_ its off-diagonal variant (diagonal set to zero; see Section 3.3). We define $A_{F}^{\text{off}}(C)={\left\|C_{0}-C_{0}^{⊤}\right\|}_{F}/{\left\|C_{0}\right\|}_{F}$. Channels with near-deterministic rows (e.g., collapsed responses) are flagged and excluded based on entropy and response dominance criteria computed from *C* (Appendix A2.1). We then estimate asymmetry–RD relationships via rank correlations, block-demeaned interaction models, and accuracy-controlled regressions; full specification is in Appendix A2.2.

### 3.5 Generative Simulation Linking Asymmetric Inductive Bias to RD Signatures

Our empirical results quantify asymmetry and RD geometry but do not expose the generative mechanisms behind their relationship. We therefore simulate systems with tunable asymmetric distortion structures to test interpretability and recoverability. Specifically, we predict that broad—weak and sink-like asymmetry organizations will produce opposite effects on RD geometry. Distributed asymmetries should expand the RD frontier by preserving information across many class distinctions, while concentrated sink-like asymmetries should collapse it by funneling probability mass into a few dominant responses. This dissociation should persist even after controlling for overall accuracy. We fix *K* = 16 classes. Each replicate involves a ground-truth distortion matrix *ρ*_true_, simulated confusion counts *N*, inferred distortion ρˆ, and derived metrics. We construct *ρ*_true_ = *ρ*_sym_+*aA* where *ρ*_sym_ is symmetric, *A* is skew-symmetric (*A* = -*A*^⊤^), *ρ_ii_* = 0, and *a* ≥ 0 controls antisymmetry magnitude. We evaluate two antisymmetry types: (i) *broad–weak* (dense skew-symmetric noise), and (ii) *sink-like* (targeted bias toward a small set of classes). Given *ρ*_true_, we generate channels via Blahut–Arimoto iterations, draw confusion counts from a multinomial distribution, and recover ρˆ using the same MAP pipeline as for empirical systems. Simulations sweep antisymmetry magnitude *a*, generation inverse temperature λ_gen_, and per-class trial count *N*_per row_ across both structures with multiple seeds. Full grid details, secondary diagnostics, and sensitivity checks are reported in Appendix A4 (Tables 3–5).

## 4 Results

### 4.1 Asymmetry magnitude and sparsity dissociate between humans and ANNs.

**Figure 2:**
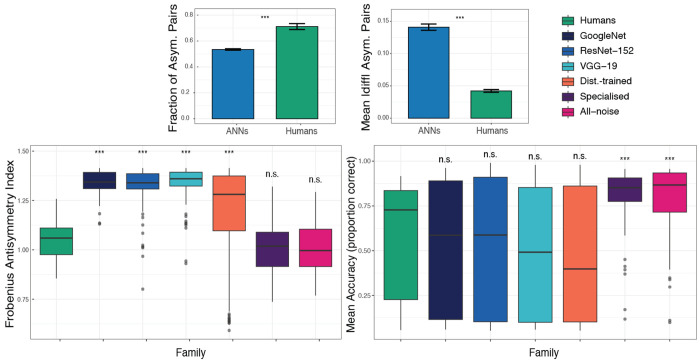
Asymmetry decomposes into *breadth* vs. *strength*, revealing a dissociation between humans and ANNs invisible to accuracy. Top: Breadth (*f*_pairs_, left) and strength (￣Δ, right) of directional structure, comparing all ANNs pooled against humans. Error bars show s.e.m. across blocks; significance marks two-sided Wilcoxon rank-sum tests. **Bottom:** Frobenius asymmetry index (left) and mean accuracy (right) by model family, each compared individually against humans (planned Wilcoxon tests, BH–FDR corrected). Each point is one block (experiment×condition×model). Groups significantly more asymmetric than humans show no accuracy difference (n.s.), and vice versa, confirming a double dissociation between asymmetry organization and accuracy.

We quantified asymmetry using block-wise summaries (one value per unique *experiment*×*condition*×*model*; ANNs: *n* = 1569 blocks; humans: *n* = 81 blocks). ANNs exhibited larger *global* asymmetry than humans (see Fig. 2) as measured by the Frobenius index (mean±SE: ANNs 1.22 ± 0.0047 vs. humans 1.04 ± 0.0097; Wilcoxon rank-sum *p* < 2.2 × 10^−16^). Despite this larger global asymmetry, ANNs showed *sparser* directional structure. Humans had more asymmetric class pairs than ANNs (ANNs 64.2 ± 0.67 vs. humans 85.4 ± 2.76; Wilcoxon *p* = 2.24 × 10^−11^), equivalently a higher fraction of asymmetric pairs (ANNs 0.535 ± 0.0056 vs. humans 0.712 ± 0.023; Wilcoxon *p* = 2.24 × 10^−11^). This sparsity gap was even larger when restricting to baseline CNNs: only baseline ANNs 54.0 ± 1.45 vs. humans 85.4 ± 2.76 (Wilcoxon *p* < 2.2 × 10^−16^). Conversely, conditional on a pair being asymmetric, ANNs showed substantially larger per-pair directional deviations (conditional mean ∆¯: ANNs 0.141 ± 0.0049 vs. humans 0.0422 ± 0.0022; Wilcoxon *p* = 6.55 × 10^−5^), revealing a dissociation between *breadth* (more pairs in humans) and *strength* (larger deviations in ANNs; Fig. 1, **Box 5**).

Planned humans vs. model comparisons of Frobenius asymmetry (BH–FDR across groups) indicated that baseline CNNs and the Distortion-trained regimes remain significantly more asymmetric than humans, whereas the specialised and all-noise regimes were not significantly different from humans (Fig. 2), suggesting that robustness-oriented training can reduce global asymmetry toward the human range (see the Appendix for full test statistics and effect-size summaries). However, as we show below, this reduction in global asymmetry does not recover the human-like breadth–strength organization, indicating that scalar asymmetry metrics are insufficient proxies for representational alignment (Fig. 1, **Box 5**). This dissociation suggests that humans and ANNs impose qualitatively different priors under distribution shift: humans distribute errors broadly across the similarity space, while ANNs concentrate failures into a small number of dominant collapse directions. Importantly, this dissociation runs in both directions (Fig. 2, bottom-right). Groups that are significantly more asymmetric than humans (GoogLeNet, ResNet-152, VGG-19, Distortion trained) show no significant difference from humans in accuracy, whereas groups that match humans on asymmetry (Specialised, All-noise) are significantly more accurate than humans. This double dissociation confirms that asymmetry structure and accuracy are genuinely independent and that directional confusion structure captures inductive bias information invisible to performance-based evaluation.

To assess generalizability beyond classic CNNs, we replicated the Frobenius asymmetry analysis on 15 modern architectures including Vision Transformers, large CNNs, and self/semi-supervised models. All families remained significantly more asymmetric than humans (all p < 0.001, BH-FDR; Fig. 5, see Appendix A3) regardless of accuracy level, confirming the dissociation is not architecture-specific.

### 4.2. Asymmetry tracks RD efficiency and curvature, above and beyond accuracy.

Asymmetry organization was systematically related to RD trade-off geometry across systems and perturbation conditions, but the strength and direction of coupling depended on model family and training regime. Crucially, naive correlations between asymmetry and RD signatures partly reflect shared dependence on accuracy: we therefore report both pooled associations and accuracy-controlled within-block regressions, isolating directional structure as a predictor above and beyond performance level (full specification in Appendix A2.2; collapsed channels excluded per Appendix A2.1).

#### Efficiency (AUC).

**Figure 3:**
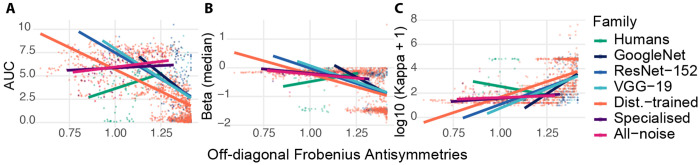
Directional confusion asymmetry covaries with RD signatures across humans and model families. Each point is one block (experiment×condition×model); *x*-axis is off-diagonal Frobenius asymmetry $A_{F}^{\text{off}}$ . Thick lines show family-wise linear trends. (**A**) Efficiency (AUC). Greater asymmetry coincides with reduced RD efficiency in most ANN families, with a distinct human profile. (**B**) Slope (*β*). Directional confusions track steepness of the information–error trade-off. (C) Curvature (log_10_ (*κ* + 1)). Regime-dependent coupling between directional structure and RD nonlinearity. All *κ* values are strictly positive (range: 8.6–3.5 × 10^9^). Apparent sub-zero region of the *y*-axis reflects extrapolation of group-wise linear trends beyond the data range.

Greater asymmetry was associated with lower RD efficiency across most ANN families, but this coupling was largely driven by shared dependence on accuracy rather than residual directional structure — with one important exception in the Distortion-trained regime (Fig. 3A). Pooled rank correlations were strongly negative for Distortion-trained models (*ρ* = −0.73, *n* = 1182) and also negative for the Baseline CNNs (GoogLeNet: *ρ* = −0.40; ResNet-152: *ρ* = −0.39; VGG-19: *ρ* = −0.43; each *n* ≈ 80). A block-demeaned interaction model (demeaning within (experiment, condition) blocks) further indicated a substantially stronger within-block asymmetry–AUC dependence for Distortion-trained models than for humans (Δslope = −7.89, *p* = 1.27×10^−7^), with an additional negative interaction for Specialised models (Δslope = −4.77, *p* = 0.014) and a directionally negative but only marginal effect for All-noise (Δslope = −3.55, *p* = 0.055). However, because asymmetry is itself tightly coupled to performance accuracy, we tested whether these patterns persist after accounting for accuracy differences within blocks. In an accuracy-controlled block-demeaned regression, the Distortion-trained models exhibited a robust *positive* conditional association between asymmetry and efficiency (accuracy-controlled within-block slope = 1.02 ± 0.12, *t* = 8.74, *p* = 6.0 × 10^−18^), while humans and baseline models showed no reliable accuracy-controlled slopes (baseline models: all *p* ≥ 0.14; humans: slope = −0.89, *p* = 0.064). This negative pooled associations largely reflects shared accuracy dependence, with regime-specific residual structure emerging only in the Distortion-trained regime after accuracy control.

#### Slope (*β*).

A similar pattern held for RD slope: asymmetry and *β* were negatively correlated across most regimes, but these associations were largely mediated by accuracy, with accuracy-independent coupling emerging only in the Specialised regime (Fig. 3B). Specifically, Distortion-trained models showed a negative marginal association (*ρ* = −0.57, *n* = 1182) and Baseline CNNs exhibited negative rank correlations (GoogLeNet: *ρ* = −0.33; ResNet-152: *ρ* = −0.46; VGG-19: *ρ* = −0.35). A block-demeaned interaction model indicated a steeper within-block dependence for Distortion-trained models than for humans (Δslope = −1.21, *p* = 0.0085), with a weaker but statistically reliable negative interaction for Specialised models (Δslope = −1.22, *p* = 0.0418) and no significant interaction for All-noise models (Δslope = −0.83, *p* = 0.142). Importantly, these effects were not uniformly robust to accuracy control. In the accuracy-controlled block-demeaned analysis, Distortion-trained models no longer showed a reliable asymmetry–*β* relationship (slope = 0.07, *p* = 0.46), while Specialised models retained a significant negative association (slope = −0.79 ± 0.39, *t* = −2.02, *p* = 0.044). Baseline CNNs again showed no reliable accuracy-controlled effects (all *p* ≥ 0.25).

#### Curvature (*κ*).

Curvature showed the most accuracy-dependent pattern of the three RD signatures. Positive associations with asymmetry in robustness-trained regimes disappeared entirely under accuracy control, suggesting that curvature primarily tracks performance rather than directional structure (Fig. 3C). Distortion-trained models showed a strong positive rank association (*ρ* = 0.72, *n* = 1182) and a significantly steeper within-block asymmetry–curvature dependence than Humans in the block-demeaned interaction model (Δslope = 3.96, *p* = 1.97 × 10^−5^). Analogous positive interaction slopes were observed for All-noise (Δslope = 2.56, *p* = 0.0256) and Specialised (Δslope = 3.40, *p* = 0.0049) models, despite Specialised exhibiting an opposite pooled rank tendency (*ρ* = −0.31). However, these effects were not robust to accuracy control. In an accuracy-controlled within-block interaction model, accuracy was strongly predictive of curvature (block-demeaned accuracy term A_dm_ = −4.22, *p* = 9.04 × 10^−14^), whereas neither the main within-block asymmetry term (demeaned asymmetry *x*_dm_ : *p* = 0.153) nor any asymmetry-by-group interaction was significant (Distortion-trained: *p* = 0.450; Specialised: *p* = 0.388; All-noise: *p* = 0.871; CNNs: all *p* ≥ 0.251).

### 4.3. Generative Simulation

We implemented the mechanistic simulation to ask the following targeted question: *When directional confusions increase, does the underlying rate–distortion (RD) geometry expand in the same way for different forms of asymmetry?* We compared two generators matched on the same control parameters (generalization regime and sample size) but differing in how directionality is organized (Fig. 1, Box 6). There was one *broad–weak* mechanism that distributes weak one-way biases across many class pairs, versus a second *sink-like* mechanism that concentrates probability mass into a small set of strong one-way errors. We report trends over all non-collapsed runs and use a strict-recovery filter only as a sensitivity check (see Appendix 3).

#### Directional structure is recoverable but identifiability is mechanism-dependent.

Across the full simulation grid (*n* = 1800 runs), numerical collapse was rare (138/1800 = 7.7%), leaving *n* = 1662 non-collapsed runs for primary analyses. A stricter reliability screen, requiring that the *recovered symmetric component* aligns with ground truth (correlation > 0.2), removed an additional 509/1662 = 30.6%, yielding *n* = 1153 strictly-recovered runs (Appendix 3). Recovery was strongly *mechanism-dependent*, with broad–weak structure exceeding sinks in pass rate in every (λ_gen_, *N*_per row_) slice and FDR significance in 10/15 slices (BH–FDR; Table 3). The largest gaps occurred at moderate-to-high generalization sharpness and intermediate-to-large sample sizes (maximum pass-rate gap 0.417; e.g. 0.867 vs 0.450), indicating that sink-like directional structure is intrinsically harder to identify under the same MAP pipeline. Consequently, we report all main trends on the *non-collapsed* set and use strict-recovery only as a sensitivity check (Appendix 3).

This differential recoverability implies that our MAP pipeline may underestimate the prevalence of sink-like structure in empirical data. Results involving sink-like regimes should therefore be interpreted with this asymmetry in mind.

#### Broad–weak versus sinks produce opposite couplings between antisymmetry and RD geometry.

**Figure 4:**
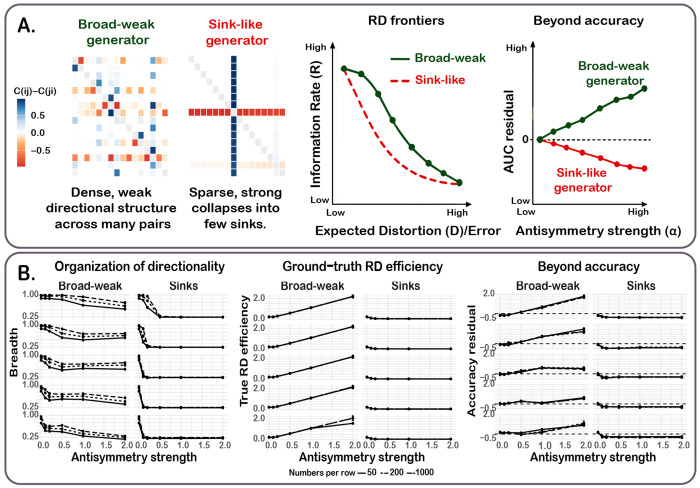
Generative simulation: asymmetry organization controls RD geometry. **A.** Left: simulated asymmetry matrices from one representative replicate per generator (*a* = 1.0, λ_gen_ = 2.0,*N* = 200). Right: schematic illustrations of the predicted RD frontier divergence and accuracy-residualized AUC, consistent with the empirical results in panel B. **B.** Full simulation grid. Columns compare generators; rows show five generalization regimes (λ_gen_ ∈ {0.2, 0.5, 1, 2, 5}, top to bottom).

**Figure 5:**
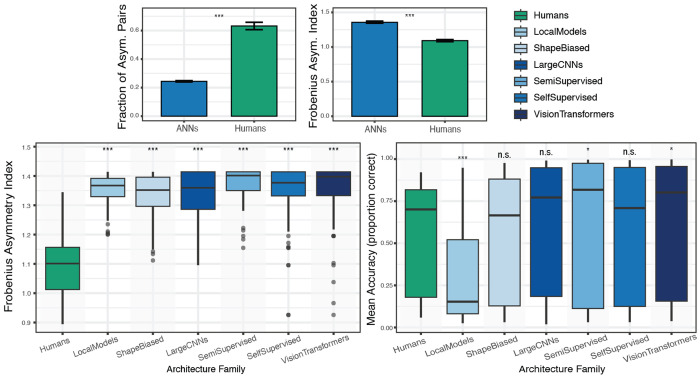
Asymmetry decomposes into *breadth* vs. *strength*, revealing a dissociation between humans and ANNs that is invisible to accuracy. Top row: Fraction of asymmetric class pairs (left) and Frobenius asymmetry index (right) comparing humans against all 15 modern model architectures. Error bars show s.e.m. and significance marks correspond to two-sided Wilcoxon rank-sum tests on block-wise summaries. **Bottom row:** Frobenius asymmetry index (left) and mean classification accuracy (right) by architecture family. Each point summarizes one block (unique experiment×condition×model). Boxes show the distribution across blocks. Significance marks are based on planned Wilcoxon comparisons of each family against humans with BH–FDR correction.

Consistent with our prediction, the same increase in antisymmetry strength produces opposite changes in RD geometry depending on whether directionality is distributed broadly or concentrated into sinks. In the broad–weak generator, increasing antisymmetry systematically *expands* ground-truth RD geometry, whereas in the sink-like generator it *collapses* ground-truth RD geometry toward a near-degenerate regime. This qualitative dissociation is visible in the true RD efficiency curves across regimes and remains consistent under strict-recovery filtering (Fig. 4; Table 4). This provides a concrete generative explanation for the empirical dissociation in which humans and ANNs can exhibit comparable global asymmetry magnitude yet occupy different regions of RD space.

#### The RD–asymmetry coupling is not reducible to overall performance.

A natural concern is that RD geometry might simply track overall performance. However, removing accuracy within each (λ_gen_ , *N*_per row_ ) slice, the dissociation persisted: broad–weak generators show strongly positive accuracy-adjusted AUC slopes across all 15 slices (all *p*_FDR_ ≤ 7.9 × 10^−31^), while sink slopes are near-zero or negative (Fig. 4; Appendix A4).

#### The breadth–strength decomposition is predictive.

To connect mechanism to observable summaries, we decomposed directional structure into *breadth* (how many class pairs exhibit directionality) and *strength* (how large the directional deviation is among asymmetric pairs). After residualizing outcomes for accuracy *within* (λ_gen_, *N*_per row_) slices, we asked which aspect of asymmetry explains residual variation in RD signatures (Appendix 4). For residual RD *efficiency* (AUC), the component model showed strong and slice-consistent effects. Breadth was typically negative (median coefficient −2.05, range [−5.40, 1.52]; significant in 11/15 slices, BH–FDR) and strength was also typically negative (median −4.28, range [−11.56, 2.45]; significant in 12/15 slices). Importantly, their interaction was typically positive (median +5.33, range [−4.65, 16.27]; significant in 5/15 slices), indicating that residual AUC depends on *how* directionality is distributed (broad-and-weak vs sparse-and-strong mixtures), not merely “more” or “less” asymmetry. In contrast, a one-number global magnitude model was weak for AUC residuals (Frobenius coefficient median −0.0047, range [−0.047, 0.206]; significant in only 3/15 slices). These results confirm that global *A*_*F*_^off^ obscures structurally meaningful organization with AUC depending on the breadth–strength decomposition, while slope and curvature track global magnitude (full regression summaries in Table 4).

## 5 Discussion

Human and ANN vision systems can achieve similar categorization accuracy while relying on fundamentally different inductive biases, differing not in how often they err, but in how failures are organized. The breadth–strength dissociation we document reflects distinct priors about which features and prototypes are privileged under uncertainty. Humans distribute errors broadly, consistent with graded similarity across many feature dimensions, while ANNs concentrate failures into a small number of dominant collapse directions. Crucially, this difference is invisible to global asymmetry magnitude. Robustness-oriented training can match scalar asymmetry metrics while leaving the underlying organizational structure unchanged, meaning two systems can appear aligned while failing for qualitatively different reasons. Treating confusion matrices as effective noisy channels and linking their directional structure to RD frontier geometry reveals why: breadth– strength organization specifically predicts how efficiently a system compresses stimulus–response information (AUC). Slope (*β*) and curvature (*κ*) by contrast primarily track the global magnitude of asymmetry rather than its organizational structure (Section A4.2). The same global asymmetry score can thus index fundamentally different generalization regimes depending on whether errors are distributed or concentrated — a distinction that accuracy-based evaluation cannot recover.

At the representational level, the breadth–strength dissociation we observe behaviorally would be consistent with differences in the geometry of learned feature spaces. Broad–weak asymmetries, as seen in humans, would be consistent with representational manifolds in which categories are arranged along graded similarity gradients (anisotropic but smoothly varying), such that many class pairs are weakly but meaningfully separated. Sink-like asymmetries, as seen in ANNs, would be consistent with representations that collapse many inputs onto a small set of dominant attractor states, producing strong but sparse directional biases. Establishing this link empirically is an important open direction. Notably, RD geometry signatures have been shown to couple directly to the structure of penultimate-layer representations [Caglar et al., 2026], suggesting that the behavioral asymmetry organization documented here may reflect, and could be used to probe, internal representational geometry without requiring direct access to activations.

Several asymmetry–RD associations nonetheless attenuate under accuracy control, indicating that naive correlations can partially reflect shared dependence on performance rather than structure alone. Our generative simulations reinforce this point by showing that the same increase in directional asymmetry can produce *opposite* RD behaviors depending on asymmetry organized. In our simulations, broad–weak asymmetries shift the RD frontier toward higher efficiency, whereas sink-like asymmetries shift the frontier toward lower efficiency. These effects persist even under accuracy control. Together, the simulations formalize a generative link between the *organization* of directional confusions and the capacity–generalization trade-offs that shape behavior. This provides a generative account of our second hypothesis, showing that the *same* asymmetry magnitude can induce different RD frontier geometry depending on whether asymmetries are broad–weak or sink-like. On a practical level, we demonstrate that matching human accuracy or aggregate asymmetry is not sufficient for human-like robustness, as systems can concentrate failures into sink-like collapses while appearing aligned on scalar metrics. A natural corrective is to penalize sink-like collapse directly as a training signal, which is computable from confusion matrices accumulated during training.

### Limitations and future directions.

We note several boundary conditions that also point toward productive extensions. First, analyses are confined to a *K* = 16-class categorization structure dictated by the GEN benchmark. Whether the breadth–strength dissociation holds at finer or coarser label granularities remains to be tested. Second, human estimates rest on *n* = 81 blocks aggregated from ~83k trials. While per-block trial counts are sufficient for asymmetry detection, the human sample is substantially smaller than the ANN sample (*n* = 1569 blocks), and replication with denser human data would strengthen the breadth–strength characterization. Third, the MAP inference of *ρ* is validated against ground truth only in simulation (Tables 3–5). Sensitivity to prior specification in the empirical setting is not directly assessed. Further open directions include class-level asymmetry analysis to identify which specific confusions drive the observed patterns, extension to adversarially-trained and certified-robust models, and application to other modalities and task domains to test whether the breadth–strength dissociation reflects a general property of biological versus artificial inductive bias or one specific to vision under distribution shift.

## Figures and Tables

**Table 1:** Block-wise asymmetry summary. One value per unique experiment×condition×model block. Values are mean±SE across blocks.

Metric	Blocks (A/H)	ANNs (mean±SE)	Humans (mean±SE)
Frobenius asymmetry index	1569 / 81	1.220 ± 0.0047	1.044 ± 0.0097
# asymmetric pairs (*n*_pairs_), all ANNs	1569 / 81	64.16 ± 0.666	85.41 ± 2.76
# asymmetric pairs (*n*_pairs_), baseline ANNs	243 / 81	53.95 ± 1.45	85.41 ± 2.76
Fraction of asymmetric pairs	1569 / 81	0.535 ± 0.0056	0.712 ± 0.023
Conditional strength $\overset{‾}{\Delta }=E_{i<j}\left[\left|C_{ij}-C_{ji}\right|||C_{ij}-C_{ji}|>\varepsilon \right]$	1569 / 81	0.1409 ± 0.0049	0.0422 ± 0.0022

**Table 2:** Asymmetry–RD associations by group. Spearman correlations between Frobenius asymmetry and RD metrics (AUC, *β*_median_, log_10_ (*κ*+1)), plus block-demeaned slope differences (∆slope) vs. humans.

(A) Rank correlations					
Group	*n*	%Coll.	*ρ*AUC	*ρβ*	*ρκ*
Humans	81	0.00	0.29	0.17	−0.22
GoogLeNet	81	0.00	−0.40	−0.33	0.43
ResNet-152	80	1.23	−0.39	−0.46	0.41
VGG-19	80	1.23	−0.43	−0.35	0.48
Distortion-trained	1182	3.11	−0.73	−0.57	0.72
Specialised	53	0.00	0.37	0.18	−0.31
All-noise	53	0.00	0.32	0.22	−0.36

(B) ∆slope vs. humans			
Group	AUC	*β*	*κ*
Humans		0 (ref.)	
GoogLeNet	2.67 (*p*=.34)	0.91 (.29)	−0.87 (.62)
ResNet-152	−0.69 (.74)	0.36 (.58)	−0.48 (.71)
VGG-19	−1.36 (.57)	−0.35 (.63)	0.61 (.68)
Distortion-trained	**−7.89** (1.3×10^−7^)^***^	−**1.21** (.0085)^**^	**3.96** (2×10^−5^)^***^
Specialised	−**4.77** (.014)^*^	−**1.22** (.042)^*^	**3.40** (.0049)^**^
All-noise	−3.55 (.055)	−0.83 (.14)	2.56 (.026)^*^

* *p* < 0.05,

** *p* < 0.01,

*** *p* < 0.001.

**Table 3:** Recovery sensitivity. A run is counted as recovered if the correlation between recovered and true symmetric confusions exceeds 0.2. The table reports recovery fractions within each (λ_gen_ , *N*_per row_) slice and BH–FDR adjusted *p*-values for differences in recovery rates (two-sample proportion tests).

λ_gen_	*N*_per row_	Frac. recovered (broad–weak)	Frac. recovered (sinks)	∆ frac.	*p*FDR
0.2	50	0.567	0.433	0.133	0.232
0.2	200	0.700	0.583	0.117	0.271
0.2	1000	0.867	0.633	0.233	0.009
0.5	50	0.667	0.517	0.150	0.172
0.5	200	**0.867**	**0.450**	**0.417**	<0.001
0.5	1000	0.817	0.483	0.333	<0.001
1.0	50	0.817	0.500	0.317	0.001
1.0	200	0.783	0.433	0.350	<0.001
1.0	1000	0.817	0.517	0.300	0.002
2.0	50	0.817	0.533	0.283	0.003
2.0	200	0.817	0.417	0.400	<0.001
2.0	1000	0.767	0.400	0.367	<0.001
5.0	50	0.900	0.517	0.383	<0.001
5.0	200	0.833	0.750	0.083	0.369
5.0	1000	0.817	0.633	0.183	0.056

**Table 4:** Residual regression summary. Within each (λ_gen_, *N*_per row_) slice, we first residualize each RD signature by mean diagonal probability (accuracy proxy), then regress the residual on either (i) breadth/strength terms or (ii) a global asymmetry magnitude term. “Structure offset” refers to contrast between sink-like and broad–weak configurations. Coefficients are summarized across slices; significance counts use BH–FDR within-slice tests.

Outcome	Predictor set	Term	Median	Min	Max	Sig. slices	Slices
AUC	components	Breadth	−2.05	−5.40	1.52	11	15
AUC	components	Strength	−4.28	−11.56	2.45	12	15
AUC	components	Breadth × Strength	5.33	−4.65	16.27	5	15
AUC	components	Structure offset	−0.447	−0.657	−0.162	15	15
AUC	magnitude	Global asymmetry mag.	−0.0047	−0.0470	0.206	3	15
AUC	magnitude	Structure offset	−0.482	−0.691	−0.171	15	15
Global slope	components	Breadth	−0.78	−2.12	0.37	3	15
Global slope	components	Strength	−1.95	−3.70	−0.40	10	15
Global slope	components	Breadth × Strength	−5.02	−9.96	5.73	7	15
Global slope	components	Structure offset	−0.188	−0.357	0.066	9	15
Global slope	magnitude	Global asymmetry mag.	−0.144	−0.202	−0.063	15	15
Global slope	magnitude	Structure offset	−0.243	−0.386	−0.081	15	15
Curvature	components	Breadth	−0.048	−0.167	0.129	3	15
Curvature	components	Strength	−0.165	−0.540	0.084	6	15
Curvature	components	Breadth × Strength	0.066	−1.030	1.250	2	15
Curvature	components	Structure offset	0.058	−0.011	0.124	7	15
Curvature	magnitude	Global asymmetry mag.	−0.201	−0.438	−0.089	15	15
Curvature	magnitude	Structure offset	0.060	−0.009	0.126	8	15

**Table 5:** Mixed-effects ANOVA key terms. Selected fixed-effect tests from replicate-level mixed models *Y* ∼ structure × *a* × λ_gen_ × log_10_ *N*_per row_ + (1|cell).

Outcome	Term	*F*	*p*
auc_true	structure	10.16	0.00146
auc_true	a_scale	1176	2.2e-16
auc_true	structure:a_scale	877.1	2.2e-16
beta_median_true_pos	structure	24.41	7.8e-07
beta_median_true_pos	a_scale	827.7	2.2e-16
beta_median_true_pos	structure:a_scale	613.4	2.2e-16
kappa_true	structure	5.34	0.0209
kappa_true	a_scale	964.7	2.2e-16
kappa_true	structure:a_scale	701.8	2.2e-16

**Table 6:** Strict-recovery robustness of residual AUC effects. For each (λ_gen_, *N*_per row_) slice and structure, we regress accuracy-residualized AUC on antisymmetry strength a under the no-collapse filter (all non-collapsed runs) and the strict-recovery filter (additionally requiring symmetric-component recovery correlation > 0.2). Columns show the slope sign and BH–FDR adjusted *p*-value under each filter, and whether the sign is consistent across filters. The qualitative pattern (broad–weak slopes positive in all 15/15 slices under both filters; sink slopes negative in all 15/15 slices) persists under strict-recovery filtering, confirming robustness to identifiability constraints.

λ_gen_	*N*_per row_	Structure	No-collapse		Strict-recovery		Match
			Sign	*p*FDR	Sign	*p*FDR	
0.2	50	Broad–weak	+	<0.001	+	<0.001	✓
0.2	50	Sinks	–	<0.001	–	0.003	✓
0.2	200	Broad–weak	+	<0.001	+	<0.001	✓
0.2	200	Sinks	–	<0.001	–	<0.001	✓
0.2	1000	Broad–weak	+	<0.001	+	<0.001	✓
0.2	1000	Sinks	–	0.002	–	0.002	✓
0.5	50	Broad–weak	+	<0.001	+	<0.001	✓
0.5	50	Sinks	–	<0.001	–	0.019	✓
0.5	200	Broad–weak	+	<0.001	+	<0.001	✓
0.5	200	Sinks	–	<0.001	–	0.042	✓
0.5	1000	Broad–weak	+	<0.001	+	<0.001	✓
0.5	1000	Sinks	–	<0.001	–	0.029	✓
1.0	50	Broad–weak	+	<0.001	+	<0.001	✓
1.0	50	Sinks	–	<0.001	–	<0.001	✓
1.0	200	Broad–weak	+	<0.001	+	<0.001	✓
1.0	200	Sinks	–	<0.001	–	0.006	✓
1.0	1000	Broad–weak	+	<0.001	+	<0.001	✓
1.0	1000	Sinks	–	<0.001	–	<0.001	✓
2.0	50	Broad–weak	+	<0.001	+	<0.001	✓
2.0	50	Sinks	–	<0.001	–	<0.001	✓
2.0	200	Broad–weak	+	<0.001	+	<0.001	✓
2.0	200	Sinks	–	<0.001	–	0.049	✓
2.0	1000	Broad–weak	+	<0.001	+	<0.001	✓
2.0	1000	Sinks	–	<0.001	–	0.012	✓
5.0	50	Broad–weak	+	<0.001	+	<0.001	✓
5.0	50	Sinks	–	0.005	–	0.012	✓
5.0	200	Broad–weak	+	<0.001	+	<0.001	✓
5.0	200	Sinks	–	0.004	–	0.011	✓
5.0	1000	Broad–weak	+	<0.001	+	<0.001	✓
5.0	1000	Sinks	–	0.012	–	0.013	✓

## A1 Compute Resources and Data Availability

### Computational resources.

All analyses are CPU-based; no GPU resources were used. The pipeline has three steps with distinct cost profiles. Step 1 - MAP inference of the latent distortion matrix *ρ* - is the computational bottleneck, scaling as *O*(*K*^2^) in both parameter space and the inner Blahut– Arimoto loop. For *K* = 16 categories, MAP inference takes approximately 50 minutes per confusion matrix on 2 CPU cores, the full empirical analysis covers 1,650 confusion matrices (all models and conditions in the GEN benchmark repository), corresponding to approximately 1,375 core-hours in total. All jobs were parallelized across an HPC cluster, significantly reducing wall-clock time. Steps 2 and 3 - RD frontier tracing and signature extraction (*β*, *κ*, AUC) - run in seconds given *ρ* and add negligible overhead. The generative simulation grid (1,800 runs across two asymmetry structures) requires approximately 10–20 CPU-hours and was likewise parallelized.

#### Data and model licenses.

The GEN benchmark [Geirhos et al., 2018b] and ModelZoo repository [Geirhos et al., 2021] are both released under the MIT License and are publicly available at https://github.com/bethgelab/model-vs-human. The human psychophysics data from both repositories is included in the same repository under the same license terms. All pre-trained CNN models (GoogLeNet, ResNet-152, VGG-19, and the robustness-trained variants) were accessed via ModelZoo and are subject to their respective original licenses. Each is cited with its original paper in the main text or the appendix. No new datasets or models are introduced in this work.

## A2 Statistical and Analytical Procedures

### A2.1 Collapsed Channel Filtering

We exclude confusion matrices with collapsed responses based on diagnostics computed from the row-normalized conditional distribution *C* (*y*|*x*) (i.e., each row of the confusion matrix normalized to sum to 1):

- Mean row entropy < 10^−3^, or
- Mean row-maximum probability > 0.999.

Only a small subset of system–condition matrices were excluded and this filtering had a negligible impact on the main trends.

### A2.2 Asymmetry–RD Regression Models

We used two linear modeling approaches to assess how asymmetry covaries with RD signatures. First, models with fixed effects per (experiment, condition) block and group-specific slopes. Second, within-block demeaning of predictor and outcome variables, followed by regression with interaction terms. Concretely, asymmetry–RD relationships are estimated via:

- *(i) Rank correlations:* Spearman correlations within group between $A_{F}^{\text{off}}$ and each RD signature.
- *(ii) Block-controlled models:* Linear models with experiment/condition fixed effects and group-specific slopes.
- *(iii) Accuracy-controlled models:* Because asymmetry and RD geometry both covary with overall accuracy, we additionally test whether asymmetry–RD associations persist after controlling for accuracy within matched (experiment, condition) blocks, isolating the contribution of directional structure independent of performance level.

## A3 Extension to Modern Architectures

### A3.1 Model selection and data.

The main analyses use matched human psychophysics and ANN behavior from the GEN benchmark repository [Geirhos et al., 2018b], which provides ~83k human trials across 12 perturbation families at graded distortion levels for *K* = 16 ImageNet-derived categories. Architecture choice in the main analysis was constrained by the requirement for matched human psychophysics data at sufficient trial density. Only models for which per-cell confusion counts are dense enough to reliably estimate directional asymmetry at the class-pair level are included. This limits the main analysis to three baseline CNNs (GoogLeNet, ResNet-152, VGG-19) and three robustness training regimes (Distortion-trained, Specialised, All-noise).

To assess whether the global asymmetry dissociation extends to modern architectures not included in the GEN repository, we additionally analyzed a broad selection of 15 architectures spanning five architecture families:

- **Large-scale supervised models**: BiT models (bitm-resnetv2-152x2, bitm-resnetv2-152x4 Kolesnikov et al. [2020])
- **Transformers and vision–language models**: Vision Transformer ViT Large Patch16-224 Dosovitskiy [2020], SWAG-ViT-L/16-IN1K Singh et al. [2022], CLIP Radford et al. [2021]
- **Architectural inductive-bias probes (LocalModels)**: BagNet-9 and BagNet-33 Brendel and Bethge [2019]
- **Robustness/shape-biased models**: standard ResNet-50 CNNs trained on Stylized ImageNet (ResNet50 SIN) and a mixture of Stylized and regular ImageNet ResNet50 (SIN+IN) Geirhos et al. [2018a]
- **Data rich semi-supervised models**: semi-weakly supervised pretrained ResNeXt-101 32x16d SWSL Mahajan et al. [2018], Noisy Student pretraining (efficientnet-l2-noisy-student-475 Xie et al. [2020])
- **Self-supervised / contrastive models**: Simple Framework for Contrastive Learning models (SimCLR resnet50x1, SimCLR resnet50x4 Chen et al. [2020a]), MoCo v2 Chen et al. [2020b], InfoMin Tian et al. [2020]

Model outputs were obtained from the ModelZoo repository Geirhos et al. [2021] using the same stimulus set, enabling direct comparison of asymmetry structure under identical perturbation conditions, with a second set of human data (~85k psychophysics trials).

### A3.2 Analytical approach and sparsity caveat

Asymmetry metrics are computed identically to the main analysis (Section 3.3), using block-wise summaries (one value per unique experiment×condition×model) on row-normalized confusion matrices. Group comparisons use two-sided Wilcoxon rank-sum tests with BH–FDR correction across planned comparisons against humans.

An important limitation of the ModelZoo repository data is that confusion matrices are sparser than in the GEN repository, since these models were not evaluated with matched per-stimulus trial counts, resulting in fewer observations per confusion matrix cell. The GEN repository confusion matrices contain on average 85 non-zero asymmetric class pairs out of 120 possible for humans (*K* = 16; 71%) and 64 for ANNs (54%), providing sufficient density for the breadth–strength decomposition. By contrast, the ModelZoo ANN confusion matrices contain on average only 29 pairs (24%), less than half the GEN ANN coverage despite identical *K* = 16 categories and stimulus conditions. ModelZoo human data achieves comparable coverage to the GEN benchmark (76 pairs; 63%) confirming that the drop in pair coverage is specific to the ModelZoo ANN confusion matrices. This sparsity simultaneously deflates estimates of asymmetry breadth (*f*_pairs_), because many off-diagonal cells are exactly zero, and inflates conditional magnitude (∆), because only the largest asymmetries survive the sparsity filter. These two metrics should therefore be interpreted with caution for the ModelZoo data. The Frobenius asymmetry index, which aggregates over all off-diagonal entries and is substantially less sensitive to the proportion of zero cells, is robust to this issue and is therefore the primary metric reported here.

### A3.3 Results

All 15 ModelZoo architecture families remain significantly more asymmetric than humans on the Frobenius index (all *p* < 0.001, BH–FDR corrected; Appendix Fig. 5, bottom-left), replicating the global asymmetry dissociation observed in the main analysis across a substantially broader range of architectures. This result holds regardless of training paradigm, including self-supervised (SimCLR, MoCoV2, InfoMin), semi-supervised (ResNeXt101-SWSL, EffNet-L2), shape-biased (ResNet50-SIN), and large-scale supervised models (BiT-M). Critically, it also holds for Vision Transformers and CLIP, which represent a qualitatively different architectural class from the CNNs evaluated in the main analysis.

The accuracy profile across architecture families is heterogeneous (**Fig. A1**, bottom-right). Local Models (BagNet-9, BagNet-33) are significantly less accurate than humans, while Vision Transformers (CLIP, ViT-L/16, SWAG ViT-L/16) are significantly more accurate. The majority of families are not significantly different from humans in accuracy. Yet all families are significantly more asymmetric than humans, confirming a double dissociation between asymmetry structure and accuracy that extends beyond the main analysis to modern architectures.

Subsequent analyses in the main paper only use the GEN data, which provides denser confusion matrices enabling reliable breadth–strength decomposition, and directly matched human psychophysics under identical stimulus conditions - both of which are necessary for the full RD geometry linkage and accuracy-controlled analyses reported in Sections 3.1 and 3.2.

## A4 Secondary diagnostics

### A4.1 RD Signatures: Global vs. Local.

We summarize RD geometry from the inferred costs using two complementary notions of *slope*:

1. **Global RD slope and curvature (primary)**. For each cost matrix (*ρ*_true_ and *ρ*) we compute an RD curve by sweeping an inverse-temperature parameter λ over a fixed grid and recording (*R*(λ), *D*(λ)). We define the global slope signature *β* (defined as the median finite-difference derivative along the frontier, *β* = median{∆*R*/∆*D*}. We define curvature *κ* as the variance of these finite-difference slopes, and efficiency (AUC) as the trapezoidal area under the RD curve. A small fraction of blocks (*n* = 25 out of 1650, occurring primarily in the Distortion-trained regime at near-chance distortion levels) yield positive *β* values. These arise when the BA sweep produces a numerically non-monotone frontier segment — an expected failure mode at extreme distortion where the confusion matrix approaches a uniform channel and the fitted cost matrix ρˆ is poorly identified. These blocks are retained in all reported correlations. Their prevalence is negligible and results are unchanged when they are excluded.
2. **Local operating-point slope (secondary)**. We compute the empirical operating rate *R*^⋆^ = *I* (*X ; Y*) from the sampled confusion *counts* (using the empirical stimulus prior), then recover the local slope *s* by root-finding for the value of λ at which the optimal channel under cost *ρ* attains mutual information *R*^⋆^.

### A4.2 Simulation Results

*Channel Generation, Sampling, and RD Model Fitting.* Given *ρ*_true_ , a generation inverse temperature λ_gen_, and a uniform stimulus prior *p(x)* = 1/*K*, we generate channels via BA iterations and draw counts via

$$N_{i.}∼Multinomial\left(N_{\text{per row}},q_{\lambda_{\text{gen}}}(\cdot |x=i)\right),$$

before recovering $\overset{ˆ}{\rho }$ by applying the same MAP RD pipeline used for empirical systems (see Sec. 2.2).

#### Simulation Grid and Diagnostics.

We compute RD signatures from both *ρ*_true_ and $\overset{ˆ}{\rho }$, and compute asymmetry metrics from the corresponding induced channels/sampled confusions. Simulations are run over grids varying antisymmetry magnitude *a*, generation inverse temperature λ_gen_, and per-class trial count *N*_per row_, across both antisymmetry structures (broad–weak, sink) with multiple seeds. We reserve *β* for the empirical RD slope metric (generalization strength) and use λ to denote inversetemperature parameters in Blahut–Arimoto and simulation generation. For pairwise breadth/strength summaries we threshold asymmetric pairs at ε = 10^−6^, and for each replicate we collect RD metrics, asymmetry scores, and recovery diagnostics (results reported in the Appendix). We also fit mixed-effects models and apply BH–FDR correction across trend tests. To validate our simulation-based inference, we computed secondary diagnostics, including Laplace-smoothed asymmetry as a sensitivity check, the operating point slope *s*^⋆^ for both *ρ*_true_ and ρˆ, and the correlation between ground-truth and fitted distortion matrices for both their symmetric and antisymmetric components.

#### Recovery sensitivity and regime-wise pass-rate tests.

We report full recovery tables and perslice two-sample proportion tests (BH–FDR), confirming broad–weak > sinks pass rates in all 15 slices and FDR significance in 10/15. We additionally summarize how recovery rates vary with λ_gen_ and *N*_per row_ and emphasize that strict-recovery contrasts are conditional on identifiability, not unconditional mechanism differences.

#### Slope and curvature behave differently from AUC: magnitude is sufficient.

The residualized RD slope magnitude and curvature show a complementary pattern. For slope magnitude, global magnitude is highly predictive across all regimes (Frobenius coefficient negative in 15/15 slices; median −0.144, range [−0.202, −0.063], BH–FDR), whereas breadth is weaker and less reliable (median −0.783, significant in 3/15 slices) and strength is more consistently negative (median −1.95, significant in 10/15 slices). For curvature (log-transformed), global magnitude is again uniformly predictive (Frobenius coefficient negative in 15/15 slices; median −0.201, range [−0.438, −0.089], BH–FDR), while breadth/strength terms are inconsistent. Thus, AUC seems to be the RD summary for which the *organization* of asymmetry (breadth vs strength) matters most, whereas slope and curvature primarily reflect overall directional magnitude.

#### Finite-sample error diagnostics for fitted versus true RD geometry.

We provide regime-wise error plots showing that sample size primarily stabilizes the recovered RD *geometry* summaries (AUC) and reduces dispersion, while local/global slope-related quantities can exhibit sporadic failures that are mechanism-specific. These diagnostics motivate using non-collapsed data for primary trends and strict recovery as a conservative sensitivity analysis.

#### Saturation behavior for sink-like organization.

We quantify the rapid transition of sink-like organization into a sparse, high-strength regime by estimating the knee point in breadth decline (and corresponding saturation in global magnitude), and we report these knee estimates by (λ_gen_, *N*_per row_) slice. This analysis supports the interpretation that sink-like directionality quickly concentrates into a small set of one-way confusions and then becomes insensitive to further increases in antisymmetry strength.

#### Strict-recovery robustness of residual effects.

Finally, we repeat the key accuracy-residualized regressions under strict recovery and summarize sign/stability of the main effects in a compact table. This confirms that the qualitative pattern—AUC residuals requiring breadth–strength organization, and slope/curvature residuals tracking global magnitude—persists under conservative filtering.

## References

[R1] Arimoto Suguru. An algorithm for computing the capacity of arbitrary discrete memoryless channels. IEEE Transactions on Information Theory, 18(1):14–20, 1972.

[R2] Attarian Maria, Roads Brett D, and Mozer Michael C. Transforming neural network visual representations to predict human judgments of similarity. arXiv preprint arXiv:2010.06512, 2020.

[R3] Blahut R.. Computation of channel capacity and rate-distortion functions. IEEE Transactions on Information Theory, 18(4):460–473, 1972. doi: 10.1109/TIT.1972.1054855.

[R4] Brendel Wieland and Bethge Matthias. Approximating cnns with bag-of-local-features models works surprisingly well on imagenet. arXiv preprint arXiv:1904.00760, 2019.

[R5] Caglar Leyla Roksan, Mediano Pedro A. M., and Lin Baihan. Rate-distortion signatures of generalization and information trade-offs, 2026. URL https://arxiv.org/abs/2603.01568.

[R6] Chen Ting, Kornblith Simon, Norouzi Mohammad, and Hinton Geoffrey. A simple framework for contrastive learning of visual representations. In International conference on machine learning, pages 1597–1607. PmLR, 2020a.

[R7] Chen Xinlei, Fan Haoqi, Girshick Ross, and He Kaiming. Improved baselines with momentum contrastive learning. arXiv preprint arXiv:2003.04297, 2020b.

[R8] D’Amato Leo, Lancia Gian Luca, and Pezzulo Giovanni. The geometry of efficient codes: How rate-distortion trade-offs distort the latent representations of generative models. PLOS Computational Biology, 21(5):e1012952, 2025. 40354307 10.1371/journal.pcbi.1012952 PMC12068621

[R9] Dosovitskiy Alexey. An image is worth 16x16 words: Transformers for image recognition at scale. arXiv preprint arXiv:2010.11929, 2020.

[R10] Geirhos Robert, Rubisch Patricia, Michaelis Claudio, Bethge Matthias, Wichmann Felix A, and Brendel Wieland. Imagenet-trained CNNs are biased towards texture; increasing shape bias improves accuracy and robustness. In International conference on learning representations, 2018a.

[R11] Geirhos Robert, Temme Carlos RM, Rauber Jonas, Schütt Heiko H, Bethge Matthias, and Wichmann Felix A. Generalisation in humans and deep neural networks. Advances in neural information processing systems, 31, 2018b.

[R12] Geirhos Robert, Jacobsen Jörn-Henrik, Michaelis Claudio, Zemel Richard, Brendel Wieland, Bethge Matthias, and Wichmann Felix A. Shortcut learning in deep neural networks. Nature Machine Intelligence, 2(11):665–673, 2020.

[R13] Geirhos Robert, Narayanappa Kantharaju, Mitzkus Benjamin, Thieringer Tizian, Bethge Matthias, Wichmann Felix A, and Brendel Wieland. Partial success in closing the gap between human and machine vision. Advances in Neural Information Processing Systems, 34:23885–23899, 2021.

[R14] Getty David J, Swets John A, Swets Joel B, and Green David M. On the prediction of confusion matrices from similarity judgments. Perception & Psychophysics, 26(1):1–19, 1979.

[R15] Gupta Shashi Kant, Zhang Mengmi, Wu Chia-Chien, Wolfe Jeremy, and Kreiman Gabriel. Visual search asymmetry: Deep nets and humans share similar inherent biases. Advances in neural information processing systems, 34:6946–6959, 2021. 36062138 PMC9436507

[R16] He Kaiming, Zhang Xiangyu, Ren Shaoqing, and Sun Jian. Deep residual learning for image recognition. In Proceedings of the IEEE conference on computer vision and pattern recognition, pages 770–778, 2016.

[R17] Ilyas Andrew, Santurkar Shibani, Tsipras Dimitris, Engstrom Logan, Tran Brandon, and Madry Aleksander. Adversarial examples are not bugs, they are features. Advances in neural information processing systems, 32, 2019.

[R18] Jakob Anthony MV and Gershman Samuel J. Rate-distortion theory of neural coding and its implications for working memory. Elife, 12:e79450, 2023. 37435811 10.7554/eLife.79450 PMC10353860

[R19] Kahana Michael J and Sekuler Robert. Recognizing spatial patterns: A noisy exemplar approach. Vision research, 42(18):2177–2192, 2002. 12207978 10.1016/s0042-6989(02)00118-9

[R20] Kolesnikov Alexander, Beyer Lucas, Zhai Xiaohua, Puigcerver Joan, Yung Jessica, Gelly Sylvain, and Houlsby Neil. Big transfer (bit): General visual representation learning. In European conference on computer vision, pages 491–507. Springer, 2020.

[R21] Liu Minghao, Wei Jiaheng, Liu Yang, and Davis James. Human and AI perceptual differences in image classification errors. In Proceedings of the AAAI Conference on Artificial Intelligence, volume 39, pages 14318–14326, 2025.

[R22] Mahajan Dhruv, Girshick Ross, Ramanathan Vignesh, He Kaiming, Paluri Manohar, Li Yixuan, Bharambe Ashwin, and Laurens Van Der Maaten. Exploring the limits of weakly supervised pretraining. In Proceedings of the European conference on computer vision (ECCV), pages 181–196, 2018.

[R23] Miller John P, Taori Rohan, Raghunathan Aditi, Sagawa Shiori, Koh Pang Wei, Shankar Vaishaal, Liang Percy, Carmon Yair, and Schmidt Ludwig. Accuracy on the line: on the strong correlation between out-of-distribution and in-distribution generalization. In International conference on machine learning, pages 7721–7735. PMLR, 2021.

[R24] Nosofsky Robert M. Attention, similarity, and the identification–categorization relationship. Journal of experimental psychology: General, 115(1):39, 1986. 2937873 10.1037//0096-3445.115.1.39

[R25] Nosofsky Robert M. Stimulus bias, asymmetric similarity, and classification. Cognitive Psychology, 23(1):94–140, 1991.

[R26] Radford Alec, Jong Wook Kim Chris Hallacy, Ramesh Aditya, Goh Gabriel, Agarwal Sandhini, Sastry Girish, Askell Amanda, Mishkin Pamela, Clark Jack, Learning transferable visual models from natural language supervision. In International conference on machine learning, pages 8748–8763. PmLR, 2021.

[R27] Recht Benjamin, Roelofs Rebecca, Schmidt Ludwig, and Shankar Vaishaal. Do ImageNet classifiers generalize to ImageNet? In International conference on machine learning, pages 5389–5400. PMLR, 2019.

[R28] Rosch Eleanor. Cognitive representations of semantic categories. Journal of Experimental Psychology: General, 104(3):192–233, 1975.

[R29] Rosch Eleanor and Mervis Carolyn B. Family resemblances: Studies in the internal structure of categories. Cognitive psychology, 7(4):573–605, 1975.

[R30] Shah Harshay, Tamuly Kaustav, Raghunathan Aditi, Jain Prateek, and Netrapalli Praneeth. The pitfalls of simplicity bias in neural networks. Advances in Neural Information Processing Systems, 33:9573–9585, 2020.

[R31] Shankar Vaishaal, Roelofs Rebecca, Mania Horia, Fang Alex, Recht Benjamin, and Schmidt Ludwig. Evaluating machine accuracy on ImageNet. In International Conference on Machine Learning, pages 8634–8644. PMLR, 2020.

[R32] Shannon Claude E. A mathematical theory of communication. The Bell system technical journal, 27 (3):379–423, 1948.

[R33] Shepard Roger N. Attention and the metric structure of the stimulus space. Journal of mathematical psychology, 1(1):54–87, 1964.

[R34] Shepard Roger N. Toward a universal law of generalization for psychological science. Science, 237 (4820):1317–1323, 1987. 3629243 10.1126/science.3629243

[R35] Simonyan Karen and Zisserman Andrew. Very deep convolutional networks for large-scale image recognition. arXiv preprint arXiv:1409.1556, 2014.

[R36] Sims Chris R. Efficient coding explains the universal law of generalization in human perception. Science, 360(6389):652–656, 2018. 29748284 10.1126/science.aaq1118

[R37] Singh Mannat, Gustafson Laura, Adcock Aaron, de Freitas Reis Vinicius, Gedik Bugra, Kosaraju Raj Prateek, Mahajan Dhruv, Girshick Ross, Dollár Piotr, and Van Der Maaten Laurens. Revisiting weakly supervised pre-training of visual perception models. In Proceedings of the IEEE/CVF Conference on Computer Vision and Pattern Recognition, pages 804–814, 2022.

[R38] Sorscher Ben, Ganguli Surya, and Sompolinsky Haim. Neural representational geometry underlies few-shot concept learning. Proceedings of the National Academy of Sciences, 119(43): e2200800119, 2022. 10.1073/pnas.2200800119 PMC9618072 36251997

[R39] Szegedy Christian, Liu Wei, Jia Yangqing, Sermanet Pierre, Reed Scott, Anguelov Dragomir, Erhan Dumitru, Vanhoucke Vincent, and Rabinovich Andrew. Going deeper with convolutions. In Proceedings of the IEEE conference on computer vision and pattern recognition, pages 1–9, 2015.

[R40] Tian Yonglong, Sun Chen, Poole Ben, Krishnan Dilip, Schmid Cordelia, and Isola Phillip. What makes for good views for contrastive learning? Advances in neural information processing systems, 33:6827–6839, 2020.

[R41] Tversky Amos. Features of similarity. Psychological review, 84(4):327, 1977.

[R42] Tversky Amos and Gati Itamar. Similarity, separability, and the triangle inequality. Psychological review, 89(2):123, 1982. 7089125

[R43] Wei Xue-Xin and Woodford Michael. Representational geometry explains puzzling error distributions in behavioral tasks. Proceedings of the National Academy of Sciences, 122(4):e2407540122, 2025. 10.1073/pnas.2407540122 PMC11789072 39854237

[R44] Xie Qizhe, Luong Minh-Thang, Hovy Eduard, and Le Quoc V. Self-training with noisy student improves imagenet classification. In Proceedings of the IEEE/CVF conference on computer vision and pattern recognition, pages 10687–10698, 2020.

